# Electrical, Piezoresistive and Electromagnetic Properties of Graphene Reinforced Cement Composites: A Review

**DOI:** 10.3390/nano11123220

**Published:** 2021-11-27

**Authors:** Shengchang Mu, Jianguang Yue, Yu Wang, Chuang Feng

**Affiliations:** College of Civil Engineering, Nanjing Tech University, Nanjing 211800, China; mushengchang@njtech.edu.cn (S.M.); jgyue@njtech.edu.cn (J.Y.); wy153472511@outlook.com (Y.W.)

**Keywords:** graphene, cement composite, electrical conductivity, piezoresistivity, electromagnetic shielding

## Abstract

Due to their excellent combination of mechanical and physical properties, graphene and its derivatives as reinforcements have been drawing tremendous attention to the development of high-performance and multifunctional cement-based composites. This paper is mainly focused on reviewing existing studies on the three material properties (electrical, piezoresistive and electromagnetic) correlated to the multifunction of graphene reinforced cement composite materials (GRCCMs). Graphene fillers have demonstrated better reinforcing effects on the three material properties involved when compared to the other fillers, such as carbon fiber (CF), carbon nanotube (CNT) and glass fiber (GF). This can be attributed to the large specific surface area of graphene fillers, leading to improved hydration process, microstructures and interactions between the fillers and the cement matrix in the composites. Therefore, studies on using some widely adopted methods/techniques to characterize and investigate the hydration and microstructures of GRCCMs are reviewed and discussed. Since the types of graphene fillers and cement matrices and the preparation methods affect the filler dispersion and material properties, studies on these aspects are also briefly summarized and discussed. Based on the review, some challenges and research gaps for future research are identified. This review is envisaged to provide a comprehensive literature review and more insightful perspectives for research on developing multifunctional GRCCMs.

## 1. Introduction

Cement composite materials (CCMs) are the most commonly used building materials in civil engineering because of their easy processability, availability, low cost, and excellent compressive strength [1]. However, CCMs also have drawbacks, such as low tensile strength, brittleness, and susceptibility to a harsh environment [2,3,4]. These drawbacks could lead to inferior durability and high cost for maintenance, which have raised great concern in civil engineering. One of the attempts to solve the above-mentioned issues is to add reinforcements into the traditional CCMs. Various fillers have been used as reinforcements for CCMs, including carbon fiber (CF) [5,6,7,8], glass fiber (GF) [9,10], carbon black (CB) [11,12], and carbon nanotube (CNT) [13,14,15]. Recently, graphene and its derivatives have been attracting great attention as reinforcing fillers for developing CCMs. Such interest stems from the extraordinary mechanical and physical properties of these nano fillers. The Young’s modulus, thermal conductivity, and electrical conductivity of the graphene fillers can reach up to 1 TPa, 5000 W·m^−1^·K^−1^ and 6000 S·cm^−1^, respectively [16,17,18]. Research has demonstrated that the addition of graphene fillers into cement matrices can remarkably improve both the physical and mechanical properties of the CCMs. For example, Najafishad et al. [19] found the compressive strength of CCMs was enhanced by 41% when 0.1 wt% graphene oxide (GO) was added. Pisello et al. [20] compared four nano-inclusions and found that graphene nanoplatelet (GNP) was the most effective nano-inclusion to improve the thermal conductivity of CCMs, enhancing the value from 0.78 W·m^−1^·K^−1^ to 1.14 W·m^−1^·K^−1^. There are many other works on the mechanical and thermal properties of graphene reinforced cement composite materials (GRCCMs) [21,22,23,24,25,26,27,28,29,30,31,32].

In addition to mechanical properties, existing studies also demonstrated that GRCCMs have considerably excellent physical properties, which stem from the inherent attributes of the graphene fillers. Utilizing these physical properties, these CCMs infused with graphene filler can be served as sensors and electromagnetic shielding materials for structural health monitoring and corrosion protection [33,34,35]. There have been a few papers summarizing the work on GRCCMs, particularly on mechanical properties. However, relatively less work has been found on comprehensive surveys of physical properties. Therefore, in this paper, we are going to review the studies that are related to three properties, i.e., electrical conductivity/resistivity, piezoresistivity, and electromagnetic shielding. Firstly, we review the materials used and the preparation methods for GRCCMs. The work on characterizing the GRCCMs by some widely used methods/facilities is introduced. Then both the theoretical and experimental work on the three properties as mentioned are presented and discussed.

## 2. Graphene-Reinforced Cement Composite Materials

The graphene fillers, cement matrices, and the preparation methods of GRCCMs are introduced and discussed, respectively, in this section.

### 2.1. Graphene Filler

**Figure 1:**
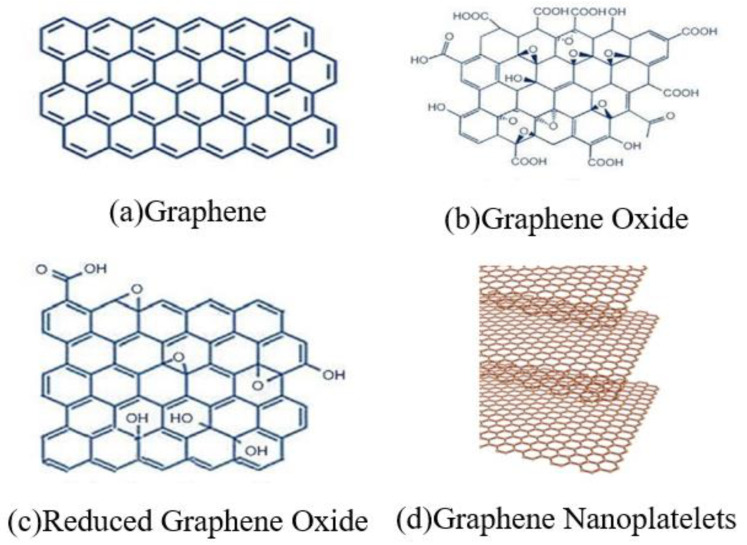
Four types of graphene fillers for GRCCMs. (**a**) Graphene, (**b**) Graphene Oxide, (**c**) Reduced Graphene Oxide. Reprinted with permission from Ref. [38]. Copyright 2021 Elsevier. (**d**) Graphene Nanoplatelets. Reprinted with permission from Ref. [39]. Copyright 2021 Elsevier.

There are different forms and types of graphene and its derivatives [36,37]. As reinforcements in a matrix, various graphene fillers have been used to develop high-performance and multifunctional CCMs. In the following, the four main types of graphene fillers as shown in Figure 1 are introduced and discussed.

(a) Graphene

**Figure 2:**
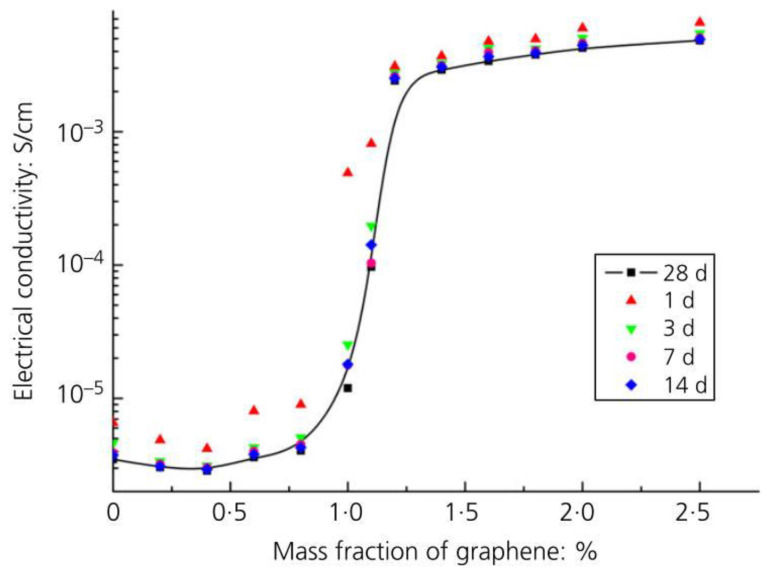
Variation of electrical conductivity of GRCCMs with graphene concentration. Reprinted with permission from Ref. [43]. Copyright 2020 Thmas Telford Ltd.

As the reinforcing filler in a matrix, it should be noted that graphene here normally refers to the sheet with a few layers instead of monolayer graphene that exists on a substrate only. It can be manufactured by mechanical, liquid-phase, and electrochemical exfoliation and chemical vapor deposition [40,41]. Extensive work has been carried out to enhance the material properties of CCMs by utilizing graphene [42,43,44,45,46,47]. Ho et al. [42] observed that the incorporation of 0.07 wt% pristine graphene increased the tensile strength of the CCMs by 26.9% at 28 days. Figure 2 shows the variation of the electrical conductivity of GRCCMs with graphene (less than three layers) concentration [43]. The electrical conductivity of the GRCCMs underwent percolation behaviour and reached a value larger than 10^−3^ S/cm.

Despite graphene’s attractive attributes, it has one obvious disadvantage—a low dispersibility—which can lead to agglomeration in the matrix. Moreover, the current methods are still challenging to massively produce high-quality graphene with moderate cost. Such a disadvantage gives rise to the other derivatives, including GO, reduced graphene oxide (rGO), and GNP.

(b) Graphene Oxide

**Figure 3:**
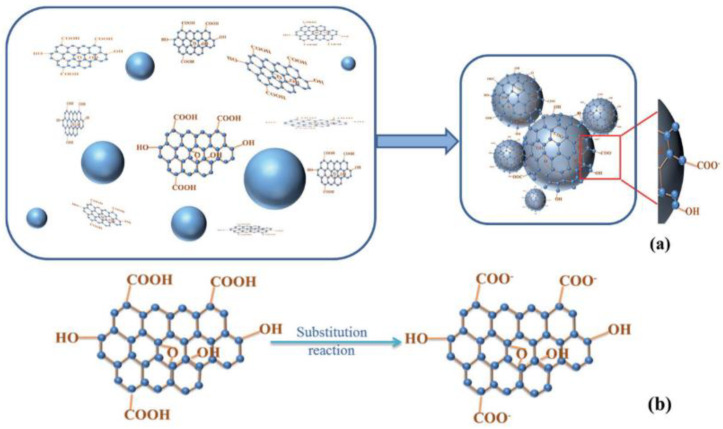
Chemical adsorption reaction schematic of GO on cement surface. (**a**) Adsorption of GO on the cement surface. (**b**) The substitution reaction of GO in the adsorption system. Reprinted with permission from Ref. [51]. Copyright 2016 RSC Publishing.

GO is a derivative of graphene with layer spacing from 0.7 to 1.2 nm. The synthesis of GO can be mainly achieved by treating graphite with oxidants and potassium permanganate followed by exfoliation. The surface of GO contains a large number of functional groups, including carbonyl, epoxide, hydroxyl, and carboxyl. Because of the functional groups, GO is hydrophilic and highly dispersible in organic solvents. This enables GOs to be the preferred reinforcing fillers for CCMs. Yu and Wu’s [48] experiments demonstrated that the addition of GO significantly increased the durability and mechanical properties of CCMs. Zhang et al.’s [49] molecular dynamic (MD) simulation showed that the incorporation of GO weakened the effects of moisture concentration on CCMs. Fan et al. [50] executed repeated simulations by MDs and found that GO and cement matrix had a strong interfacial bond strength. Wang et al. [51] reported that GO had a considerable capacity for absorption on the cement surface due to the chemical reaction of the carboxyl groups at the edge of GO filler (as shown in Figure 3).

Although GO demonstrates some advantages, it also has disadvantages. It contains defects and its mechanical properties are considerably inferior to graphene. Moreover, GO has a much lower electrical conductivity and electron mobility, limiting its application as reinforcements in developing multifunctional CCMs.

(c) Reduced Graphene Oxide

**Figure 4:**
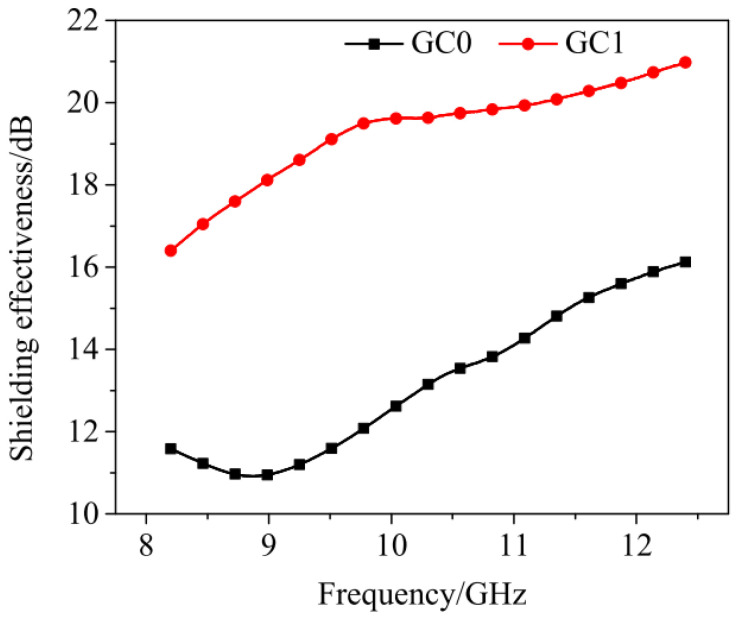
Comparison of shielding effectiveness of reinforced CCMs with and without rGO. Reprinted from Ref. [53].

rGO is another valuable derivative of graphene. It can be manufactured by removing part of the functional groups of GO. Several methods, including thermal, chemical, and photo-thermal reduction, can be utilized to manufacture rGO. The thermal reduction process takes place at a temperature between 300 °C–2000 °C in an environment with inert air or a reduced atmosphere. For chemical reduction, inorganic agents, such as hydroxylamine or phenyl hydrazine hydrate, are normally used. The photo-thermal reduction can be completed by a laser beam with a wavelength under 390 nm. After reduction, rGO can possess comparable mechanical and electrical properties such as graphene [52]. Phrompet et al. [31] demonstrated that the incorporation of rGO effectively improved the mechanical, thermal, and dielectric properties of CCMs. Zhang et al. [53] simply mixed rGO into cement and the electromagnetic shielding effectiveness was significantly improved. As shown in Figure 4, the experimental sample with 1.0 wt% rGO added has a value of 16–21 dB (GC1) for electromagnetic shielding effectiveness, an enhancement of 30% to 45% compared to the sample without rGO (GC0).

Compared to GO, rGO has favorable attributes. However, rGO also has some drawbacks. Its dispersibility in CCMs decreases compared to GO due to its hydrophobic attributes. Despite the decreased dispersibility, rGO is still regarded as a promising reinforcing filler for its excellent mechanical and physical properties as well as its low cost and availability for scalable preparation.

(d) Graphene Nanoplatelet

GNP is a reinforcing filler with platelet-shaped graphene sheets stacked. It can be obtained by using natural graphite via methods such as thermal shock, chemical exfoliation, plasma reactor or shear force. GNP possesses competent attributes such as lightweight, high specific surface, low density, electric and thermal conductance, excellent mechanical properties, lower cost, etc. Zohhadi et al. [54] and Zhu et al. [55] manufactured CCMs by using GNP as reinforcements. Their experimental results indicated that GNP improved the hydration process and generated more hydration products. Wang et al. [56] found that both the flexural and compressive strengths were significantly enhanced when GNP was added. Compared to the plain sample, the mechanical properties of reinforced CCM samples at 0.05 wt% GNPs showed the most improved results, with flexural and compressive strengths being enhanced by 27.4 and 4.3%, respectively, after 7 days of curing, and by 3.7% and 25.2%, respectively, after 28 days of curing.

### 2.2. Cement Matrix

In the following, different cement matrices in developing GRCCMs are introduced and discussed.

(a) Ordinary Portland Cement

Ordinary Portland Cement (OPC) is a water-hardened gel material, mixed with blended materials and an appropriate amount of gypsum. The amount of the blended materials does not exceed 15%. OPC has been extensively used as matrix to develop GRCCMs. Gong et al. [57] dispersed GO in OPC and found that the addition of 0.03 wt% GO increased the strength of CCMs by more than 40%. Ho et al. [42] used electrochemically prepared graphene as reinforcements and found that the flexural and compressive strength were enhanced by 26.9% and 34.3%, respectively, when 0.07 wt% graphene was dispersed. Li et al. [58] carried out experiments on the influence of GO on the early hydration process and mechanical properties of OPC pastes. The results showed that the strength of the GO reinforced cement paste was significantly enhanced, and the hydration rate was accelerated.

(b) Portland Pozzolana Cement (PPC)

**Figure 5:**
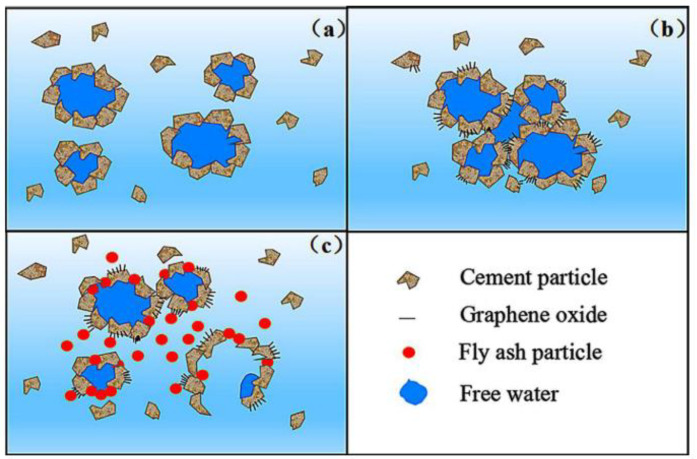
Schematic diagram of the formation of flocculation structure. (**a**) Cement paste, (**b**) GO reinforced cement paste; (**c**) GO reinforced cement paste with fly ash. Reprinted with permission from Ref. [61]. Copyright 2017 Elsevier.

PPC is a mix of OPC, gypsum, and Pozzolanic materials in a certain proportion. Slag and fly ash are usually added into OPC to develop PPC. Compared to OPC, PPC generates less hydration heat at a lower rate. It also has a low permeability. Zhu et al. [59,60] studied the effects of GO and GNP on the performances of slag PPC composites and evidenced that the fillers accelerated the hydration degree. Chintalapudi et al. [32] dispersed GO in fly ash PPC and found dense hydrated crystals formed by GO, which improved the compressive strength of the CCMs. Wang et al. [61] added GO into fly ash PPC and investigated the rheological properties. Figure 5 shows the mechanisms of the fly ash effect on the rheology of the reinforcement CCMs. The mixing of fly ash PPC and GO improved the workability of the composites. Sharma and Arora [62] used fly ash PPC as the matrix and added graphene as reinforcements. At a concentration of 0.05 vol% graphene, the flexural and compressive strength were increased by 13% and 8%, respectively. Wang et al. [63] and Saafi et al. [64] used GO and rGO, respectively, as reinforcements for fly ash PPC and found that both the fillers accelerated the secondary hydration at a later stage and reduced the porosity.

Apart from OPC and PPC, other types of cement matrices, including Magnesium Phosphate Cement (MPC), sulphoaluminate cement (SAC), and magnesium potassium phosphate cement (MKPC), are also used as matrices to develop high-performance and multifunctional GRCCMs [65,66,67,68].

### 2.3. Preparation of Graphene Reinforced Composites

**Figure 6:**
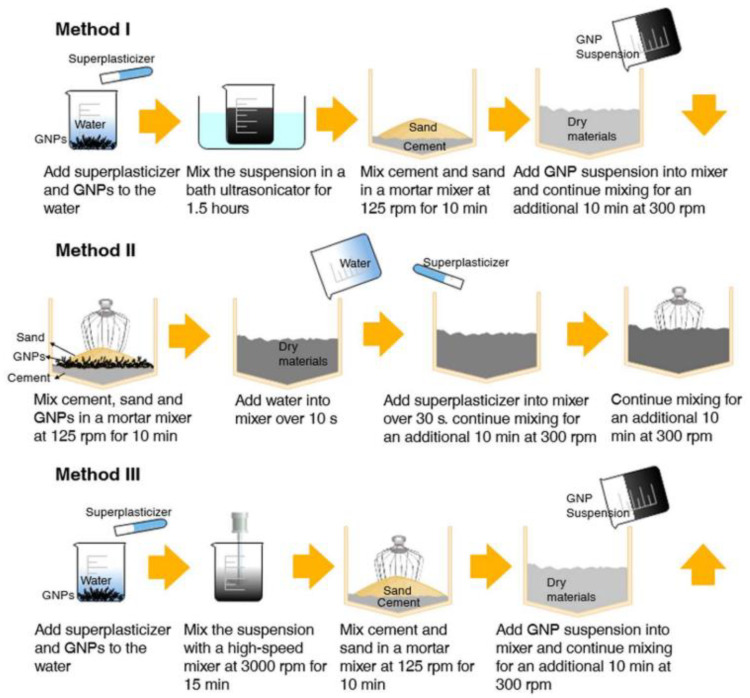
Mixing methods for graphene fillers in cement matrix. Reprinted with permission from Ref. [71]. Copyright 2018 IOP Publishing.

Due to van der Waals force, it is challenging to add graphene fillers into a cement matrix randomly and uniformly. Poor dispersion of these fillers may lead to the deterioration of the material properties and limit their engineering application. Therefore, numerous measures have been developed to enable better dispersion of graphene fillers in cement matrix. These measures can be categorized into dry-mix dispersion and wet-mix dispersion (as shown in Figure 6). Physical dispersion methods are usually used in both dry-mix and wet-mix dispersion techniques. However, it is challenging to use a physical dispersion method to disperse graphene fillers at the micro/nano meter scale [26,69,70]. Therefore, in the wet-mix dispersion, researchers combined physical with chemical dispersion methods to better disperse graphene fillers in CCMs.

#### 2.3.1. Dry-Mix Dispersion

**Figure 7:**
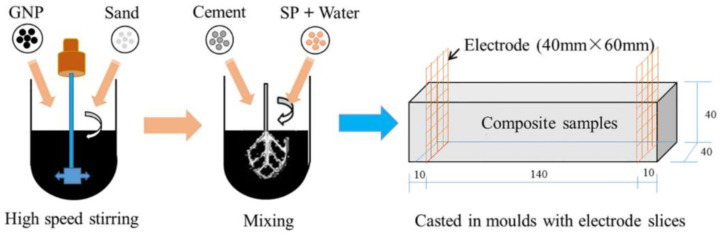
Dry-mix dispersion method to prepare GNP reinforced CCMs. Reprinted from Ref. [72]. Copyright 2018 Elsevier.

For the dry-mix method, graphene fillers and the other dry ingredients are mixed by high-speed stirring to prepare a homogeneous mixture. Then water and the other liquid ingredients are added for molding and hardening of the composites. Liu et al. [72,73] adopted the dry-mix method to prepare GNP-reinforced CCMs. As shown in Figure 7, GNP and sand were first mixed by high-speed mechanical stirring. The high-speed impact and friction between GNPs and the sand enabled the fillers to be attached to the sand and dispersed with fewer aggregates. Then cement, water and other admixtures were added subsequently and mixed. Sartipi et al. [74] mixed GO with cement and then placed the mixture on the vibrator for consolidation purposes, allowing 24 h for curing. Ghosh et al. [75] utilized a ball mill to have a homogenous distribution of GNP in cement. Mixed raw materials were placed into a cylindrical steel die for ball milling and then were compressed at 40 MPa to make it bulk and cured.

The procedure of the dry-mix dispersion method is as following: (1) the dry materials, such as graphene filler, cement, and sand, etc., are mixed by a mechanical mixer; (2) the appropriate amount of water and admixture are added into the mixture for mixing; (3) cast samples and allow them to cure [71,76]. The dry-mix dispersion method does not involve dispersing fillers in an aqueous solution. Therefore, this method does not need to consider the limitation of the water–cement ratio, which is suitable for prepare GRCCMs with a high graphene concentration.

#### 2.3.2. Wet-Mix Dispersion

Wet-mix dispersion is a technique for achieving good dispersion of graphene fillers by ultrasonication, electromagnetic/mechanical stirring, surfactant, or chemical surface modification. Then the uniform solution is mixed with a cement matrix. The wet-mix dispersion method can be categorized into physical and chemical dispersion. The physical methods in wet dispersion mainly involve ultrasonication and electromagnetic/mechanical stirring. The chemical methods involve chemical surface modification and surfactants, which usually combine with physical methods, such as the combination of surfactant/chemical surface modification and stirring, or surfactant/chemical surface modification and stirring/ultrasonication. In the following, the physical and chemical methods involved in wet-dispersion are introduced and discussed.

(a) Ultrasonication

Due to the high sound intensity, ultrasonication can stimulate a strong cavitation effect in an aqueous solution with a large number of cavitation bubbles. As these bubbles burst, a micro jet is produced which will crush the solid particles in the liquid. The solid particles and liquid can then be fully mixed. However, heat is generated during the ultrasonic process. To avoid the influence of heat, the process is usually conducted in the ice bath [77]. Prabavathy et al. [78] added rGO powder to 100 mL of a 1:1 water–ethanol mixture. Homogeneous dispersion is achieved by sonication of the mixture for approximately 30 min. The rGO suspension was aged for 6 h to evaporate ethanol and the suspension remained stable even after the aging period. Xu et al. [34] sonicated GNP in deionized water for 2 h to obtain an aqueous dispersion. The solution was then mixed with cement immediately after stirring for 10 min by using a high-speed homogenizer. Liu et al. [79] mixed polycarboxylate superplasticizer (PC) with GNPs in an aqueous solution and treated it with ultrasonication. It was found that GNPs were well dispersed in an aqueous solution after ultrasonic treatment.

(b) Electromagnetic/Mechanical Stirring

Electromagnetic stirring is mainly used for stirring and heating low viscosity liquids or solid–liquid mixtures. Akarsh et al. [80] made an aqueous solution of GO by electromagnetic stirrer at 600 to 1000 rpm. Because of the functional group, GOs were dispersed without any surfactant. Rehman et al. [81] first treated the GNP solution with ultrasonication and then stirred it for 1 h using an electromagnetic stirrer to have homogenous dispersion of the reinforcements. Then, the cement was added to the GNP suspension to fabricate experimental samples. Li et al. [58,82] used a high-speed shear mixer to prepare cement paste samples. After sonication of the graphene solution, cement powder was added, and high-speed shear mixing was performed.

(c) Surfactants

**Figure 8:**
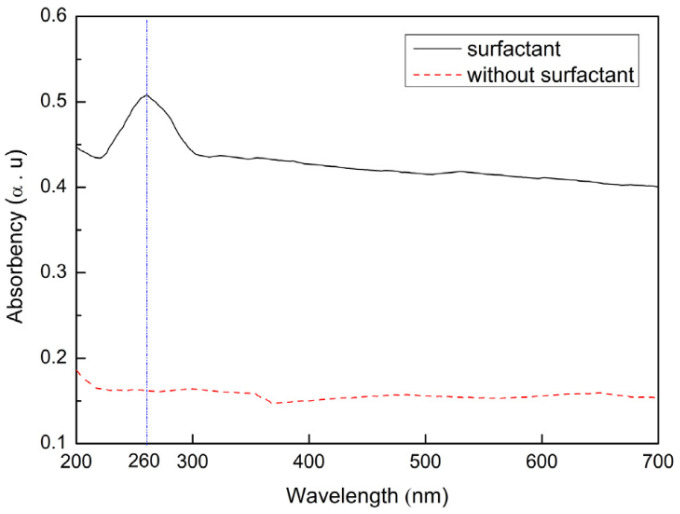
UV-Vis spectroscopy of graphene suspensions with and without surfactants. Reprinted with permission from Ref. [69]. Copyright 2018 Elsevier.

In addition to physical methods, researchers also add surfactants, such as methylcellulose (MC) [26], sodium dodecyl benzene sulfonate (SDBS) [44], lignosulfonate (LS) [83], naphthalene superplasticizer (NS) [84] and PC [83,85,86], to prepare CCMs with improved dispersion. Surfactants can maintain the intact structure of graphene filler with no damage. Surfactants can be roughly divided into ionic and non-ionic. SDBS and PC are ionic surfactants that can make graphene have good dispersion in an alkaline solution. Zhao et al. [83] used PC, LS, and polycondensate of b-naphthalene sulfonate formaldehyde (PNS) as surfactants to disperse GO in solvent. The observation indicated that PC-modified GO had improved dispersion in cement matrix compared with PNS and LS. Babak et al. [87] used PC to have a better dispersion of GO. The observation of the fracture surface of the sample indicated that GOs were well dispersed without agglomeration. Zhai et al. [88] used six dispersants in combination with ultrasonic treatment to uniformly disperse rGO into a cement base. Their experimental results indicated that NS was the best surfactant for dispersing rGO. Figure 8 shows the UV-Vis spectroscopy of two graphene suspensions with and without surfactant [69]. From the comparison, it is obvious that graphene fillers had a better dispersion in the suspension containing surfactants.

(d) Chemical Surface Modification

**Figure 9:**
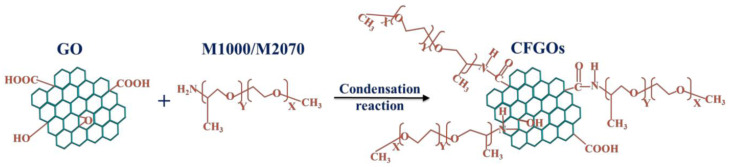
Schematic illustration of chemically functionalized GO. Reprinted from Ref. [91].

The presence of some functional groups such as hydroxyl (–OH), carboxyl (–COOH), carbonyl groups(C=O), epoxy groups (–O–), etc., on graphene and its derivatives can react with each other or some other molecules. These reactions can be used to functionalize the surface of graphene fillers with covalent bonds. Ma et al. [89] functionalized GO with NH_2_ and added the filler into cement mortar. Mercury intrusion porosimetry (MIP) found that GO and the functionalizing groups filled the pore in the CCMs. The functionalization by NH_2_ enhanced the interaction between GO and the cement matrix. Li et al. [90] prepared GO with polycarboxylic acid superplasticizer for surface functionalization. The findings of the experiment showed that the functionalized GOs were well dispersed in the CCMs. The adsorption behavior of chemically functionalized GO with three different polyether amine branched-chain lengths was studied in comparison with GO on cement by Wang et al. [91,92]. Figure 9 shows the chemically functionalized GO obtained by the condensation reaction of GO with polyether amines. The results of the experiment indicated that the chemically functionalized GO improved the dispersion.

Although chemical surface modification of graphene fillers can significantly improve their solubility and wettability and reduce agglomeration, the original excellent properties of the graphene fillers can be easily altered in the modification process.

### 2.4. Characterization

After fabrication of GRCCMs, it is evidenced that the dispersion of graphene fillers, the hydration process, and the crystallization reaction which occurred between the fillers and the cement matrix, etc., could significantly affect both the mechanical and physical properties of the GRCCMs. For example, the pore structures in the CCMs, i.e., size, distribution, and connection, are highly related to the hydration process and a reaction occurred between the graphene filler and the cement matrix. Accordingly, the investigation of the dispersion, hydration, and reaction in the composites can provide insightful information on the mechanism that underpin the reinforcing effects of the graphene fillers. Therefore, the work on using some methods/techniques, including thermal analysis, X-ray diffraction (XRD) analysis, X-ray Photoelectron Spectroscopy (XPS), Fourier-transform infrared (FTIR) spectroscopy analysis, Raman spectroscopy, Nuclear Magnetic Resonance (NMR) spectroscopy, Mercury Intrusion Porosimetry (MIP), Scanning Electron Microscopy (SEM) and Friction Force Microscopy (FFM), to characterize and investigate the GRCCMs is introduced and discussed.

#### 2.4.1. Thermal Analysis

There are mainly four methods involved for thermal analysis of GRCCMs, including thermogravimetric analysis (TGA), derivative thermogravimetric (DTG), differential thermal analysis (DTA), and differential scanning calorimetry (DSC). Thermal analysis is a method to estimate the variation of material properties with temperature and heat flux, and the hydration process of GRCCMs can be examined by the thermal analysis.

**Figure 10:**
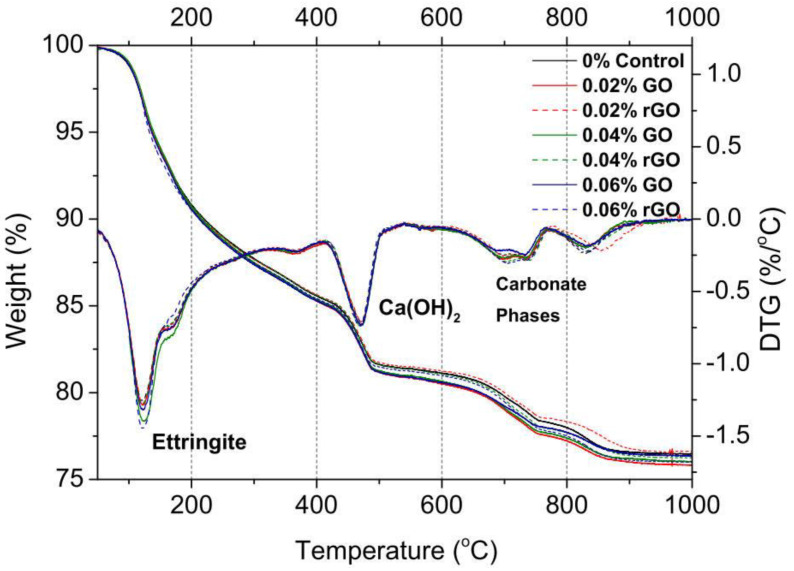
TGA and DTG curves for GO or rGO reinforced CCMs at 28 days. Reprinted with permission from Ref. [93]. Copyright 2019 Elsevier.

TGA plots the change of mass in terms of temperature while the sample is subjected to a controlled temperature. It can provide information on phase transition, absorption (desorption), thermal decomposition, etc. In contrast, DTG measures the mass loss rate (i.e., *−dm/dt*) in terms of the temperature. Compared to TGA, the DTG curve can be used to identify the critical temperature for peaks of mass loss rate. Wang et al. [26] conducted TG/DTG analysis for plain cement and GNP-reinforced CCMs at 7 and 28 days. It is found that the amounts of Ca(OH)_2_ and amorphous phases in GNP-reinforced CCMs were increased at the age of 7 days, indicating the acceleration of the hydration process. Figure 10 shows the TGA and DTG analysis for Ca(OH)_2_ decomposition in GO/rGO reinforced by CCMs by Qureshi and Panesar [93]. From the figure, it can be seen that the mass loss of GO reinforced CCMs with respect to Ca(OH)_2_ was greater than that of rGO reinforced CCMs. This indicates that rGO may have a better reinforcing effect compared to GO.

**Figure 11:**
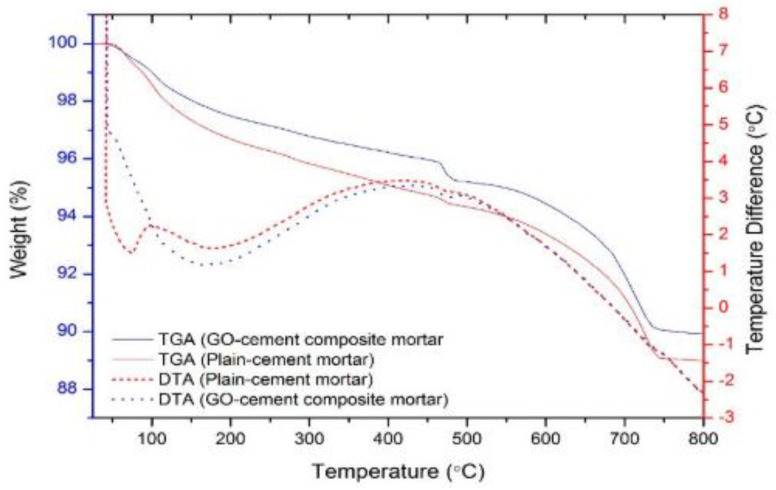
TGA/DTA curves of plain cement and GO/cement composites. Reprinted from Ref. [94].

Unlike TGA and DTG measuring temperatures directly, DTA measured the temperature difference between a sample and a thermally inert reference material when both the above two materials are undergoing a programmed temperature. This analysis can provide information on transformations like crystallization, glass transitions, sublimation, melting, etc. Sardar et al. [94] determined the properties of GO/cement composites at high temperatures using the TGA/DTA method. From the DTA curves, as shown in Figure 11, it can be identified that water loss from carboaluminate hydrate and C-S-H occurred at 180–300 °C, dehydroxylation of portlandite (calcium hydroxide) at 430–480 °C, and decarbonation of calcium carbonate at 600–780 °C. Using the TG-DTA curve, Wang et al. [95] identified that the dispersion of GO had a limited influence on the number of hydration products of the cement composite at 3 days, while at 90 days, the addition of 0.01 wt% GO promoted the hydration.

**Figure 12:**
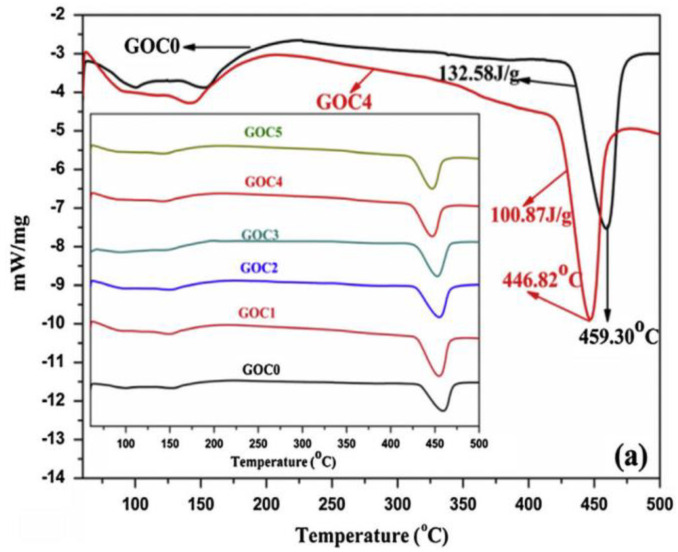
DSC curve of GO/cement composites at 28 days. Reprinted with permission from Ref. [96]. Copyright 2016 Elsevier.

Instead of measuring the temperature difference, DSC measures the difference in heat (energy) needed to keep both the reference material and the sample at the same temperature. It is an indication of the occurrence of glass transition, crystallization, oxidation, and chemical reactions. Wang et al. [96] used DSC to study the mechanisms of the effects of GO on CCMs from the hydration perspective. As shown in Figure 12, the endothermic peak at 457 °C denoted the decomposition of Ca(OH)_2_. As the GO contents in the modified cement increased, the characteristic peak generated by the decomposition of Ca(OH)_2_ dropped. Such an observation suggests that the addition of GO decreased the temperature for the decomposition of Ca(OH)_2_ and affects the hydration process. However, Wang et al. [97] conducted TG-DSC analysis on GO/cement composites at 28 days to study the hydration reaction and found that GO had limited effects on the hydration process. The above different observations may be attributed to the preparation of the composites and the dispersion of the graphene fillers in the matrix.

#### 2.4.2. X-ray Diffraction (XRD) Analysis

To further investigate the hydration process, X-ray Diffraction analysis, which is a non-destructive method, can be used to determine the crystalline structure of GRCCMs. XRD can characterize the degree of hydration of GRCCMs by monitoring the change of the peak intensity for crystalline structure of hydration products.

**Figure 13:**
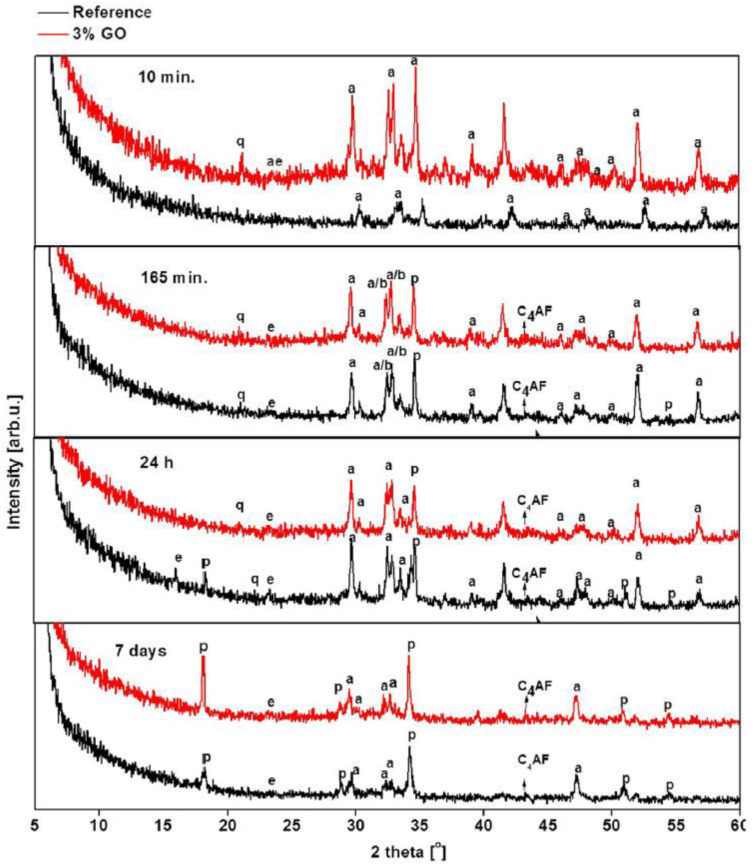
XRD result of plain cement (reference) and GO-reinforced CCMs. Reprinted with permission from Ref. [22]. Copyright 2015 Elsevier.

Horszczaruk et al. [22] used XRD to investigate the phase transition and crystalline hydration products of plain and GO-reinforced CCMs, respectively. Figure 13 demonstrates the XRD patterns for the two composite samples with different hydration times. As the hydration process continues, the intensity of the peaks attributed to un-hydrated silicates (C_3_S) and belite (C_2_S) is decreased. New peaks with respect to the formation of portlandite (Ca(OH)_2_) and ettringite (C_6_AS_3_H_32_) appeared. Since no significant difference was observed between the GO/cement and plain cement samples, the XRD results indicated that the crystal phase did not change after the addition of GO fillers.

Qureshi and Panesar [93] examined GO and rGO-reinforced CCMs at different curing times by XRD. The typical hydration products, including C_6_AS_3_H_32_, Ca(OH)_2_, CH, C_3_S, and C_2_S, were detected at all ages. The intensity of CH phases enhanced with the increases in the GO and rGO concentration and curing time. The intensities of the C_6_AS_3_H_32_ phases diminished with the hydration process and demonstrated an enhanced intensity in the rGO-reinforced CCMs. Yaseen et al. [98] employed XRD to identify the crystalline phases of the solid products. It is observed that the intensities of the CH and CaCO_3_ peaks with GO and/or rGO reinforcements were higher than the peaks without any reinforcements. The CaCO_3_ peak intensity increased with the GO and/or rGO addition. The above XRD observations suggest that the addition of GO and/or rGO can enhance the curing and hydration process in the cement composites.

#### 2.4.3. X-ray Photoelectron Spectroscopy (XPS)

XPS is a spectroscopic technique for the characterization of surfaces, which is helpful to identify the elements within or on the surface of a material, as well as their chemical bonds. XPS can be used to study the adsorption properties and chemical reactions of graphene fillers on cement and can help further determine the connection between graphene fillers and cement. This can provide information on the interaction between the graphene filler and the cement matrix.

**Figure 14:**
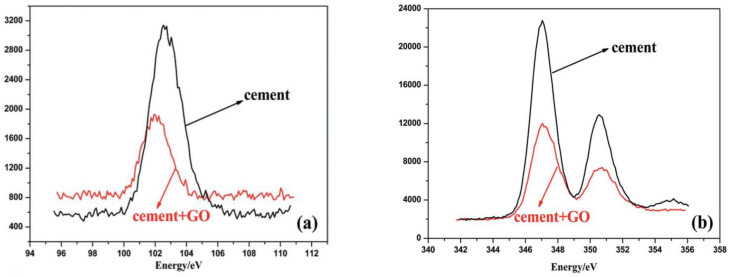
XPS results of cement surface with and without GO (**a**) Si 2p; (**b**) Ca 2p. Reprinted with permission from Ref. [51]. Copyright 2016 RSC Publishing.

Wang et al. [51,96] used XPS to study the adsorption characteristics and mechanisms of GO-modified cement composites. Figure 14 demonstrates the XPS results of the cement surface of cement with and without GO. From the figure, it can be seen that the Si 2p core-levels presented not only an intensity reduction, but also a chemical shift towards lower binding energy, indicating a reduction in the former Si-oxide (peak around 103 eV). The intensities of both Si 2p and Ca 2p decreased after the adsorption of GO nanosheets, indicating the adsorption of GO on cement. With the increase of the GO concentration, the production of Ca(HCOO)_2_ increased. Li et al. [82] also investigated the adsorption of GO fillers onto the surface of cement by XPS, in which Ca and Si were used as marker elements. Their experimental studies showed that with the addition of 0.04 wt% GO, the intensities for the two elements were unchanged, indicating very limited adsorption of GO on cement surface. Yaseen et al. [98] prepared GO and rGO-reinforced CCMs and examined the interaction during the carbonation reaction at 7 and 28 days by XPS. Because of the functional groups, the dispersion of GO resulted in a faster carbonation and a greater CaCO_3_ generation.

#### 2.4.4. Fourier-Transform Infrared (FTIR) Spectroscopy

**Figure 15:**
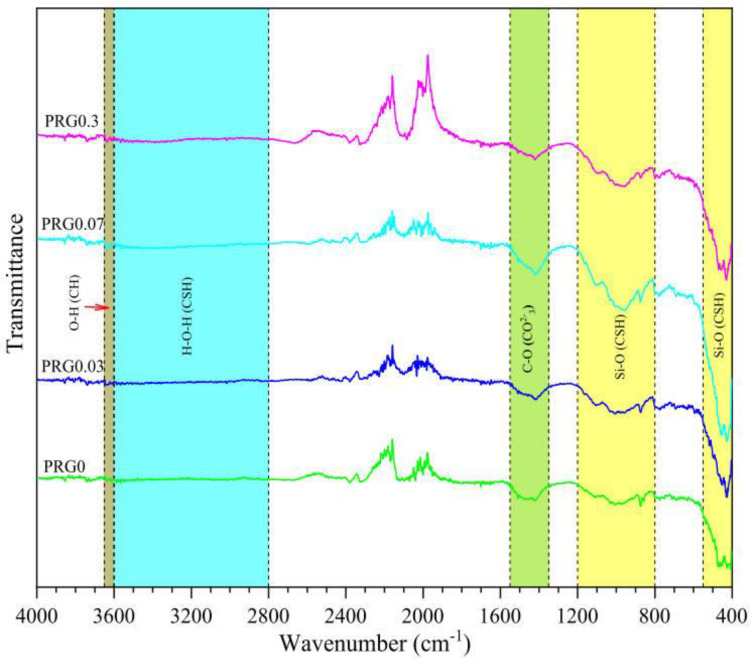
FTIR spectra of cement composites with different graphene concentrations (0%, 0.03%, 0.07% and 0.3%) at 28 days. Reprinted with permission from Ref. [42]. Copyright 2020 Elsevier.

FTIR spectroscopy is an examination method to gain the infrared spectrum of absorption or emission of a material. The spectra profile of the examined sample can be used to compare with the ones in the database to screen components in the sample. Unlike thermal analysis and XRD, FTIR can quantitatively identify the occurrence of bond stretching and interactions instead of their extent. Ho et al. [42] conducted an FTIR investigation on CCMs with different contents of graphene. As demonstrated in Figure 15, the intensities of the spectra denoting CSH gels, CH, and CaCO_3_ in GRCCMs were stronger than those of plain samples, indicating an improved enhancement in mechanical properties. This was ascribed to the facilitated hydration in the reinforced composites.

Wang et al. [51,96] conducted FTIR analysis for GO and GO-modified CCMs with different concentrations. According to the FTIR analysis, with the increase in GO concentration, the peak at ~3650 cm^−1^ corresponding to –OH in Ca(OH)_2_ became more obvious. Compared to GO-reinforced CCMs, the –COOH in GO underwent a chemical reaction to generate a new component including –COO^−^ in the GO reinforced CCMs. Yang et al. [99] compared the FTIR spectrum for GO and different cement samples. After adding GO, only a small amount of CO_3_ was generated, with no other changes occurring.

#### 2.4.5. Raman Spectroscopy

**Figure 16:**
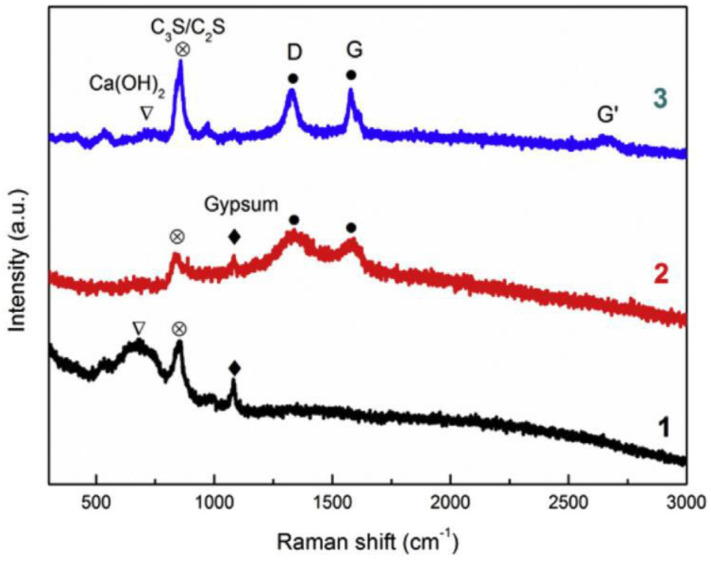
Raman results of (1) Plain cement sample; (2) GO; (3) Graphene/cement sample. Reprinted with permission from Ref. [45]. Copyright 2017 Elsevier.

Raman spectroscopy is another non-destructive analytical method that provides information on phase and polymorphy, molecular interactions, chemical structure, and crystallinity. It is a useful technique to characterize the structural configuration of GRCCMs. Hou et al. [45] used Raman spectroscopy to study the influences of the hydration reaction of cement matrix after adding graphene and GO (as shown in Figure 16). It is observed that the plain cement sample showed a distinctive Ca(OH)_2_ characteristic peak because of the hydration of C_3_S at the early stage, whereas, after the addition of GO, the peak almost vanished because of the chemical reaction of Ca^2+^ with COOH in GO to form Ca(HCOO)_2_.

Horszczaruk et al. [22] Raman spectra of GO/cement composites demonstrated two peaks (D, G). An additional band appeared between D and G, which was attributed to the formation of alite. The results showed that GO interacted well with the hydration products of Portland cement. Phrompet et al. [31] performed Raman spectra analysis for rGO/cement composites. The intensity of the D and G band peaks increased significantly with the increasing rGO contents, which confirmed that rGO combined effectively with the C3AH6 cement to form a nanocomposite.

#### 2.4.6. Nuclear Magnetic Resonance (NMR) Spectroscopy

**Figure 17:**
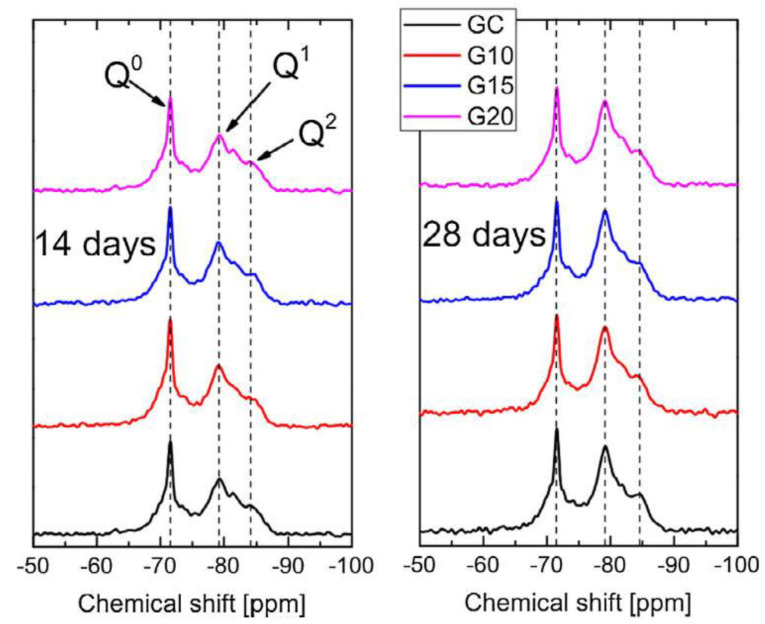
^29^Si-NMR spectra of GO/cement composite specimens at 14 and 28 days. Reprinted with permission from Ref. [99]. Copyright 2017 Elsevier.

NMR spectroscopy, which is an analytical chemistry method to obtain the purity and content, has been widely used to analyse the C-S-H structure in CCMs. Yang et al. [99] employed NMR to study the C-S-H structure in GO-reinforced CCMs at 14 and 28 days (as shown in Figure 17). Q^0^, Q^1^ and Q^2^ represented dehydrated cement, the end-chain silicate tetrahedral, and middle-chain silicate, respectively. The results showed that the hydration degree of GO-modified cement samples was higher than that of plain cement samples. Wang and Deng [55] prepared GNP/cement composites and monitored the hydration process by NMR. Their results also suggested that GNP enhanced the hydration reaction of the cement and produced more hydration products.

Xu et al. [100] performed NMR analysis of GO/cement composites and found that GO attracted Ca ions to generate jennite-like hydrates closed to GO sheets and promoted the formation of tobermorite-like hydrates far beyond GO sheets. Kang et al. [101] examined the hydration of GO/cement composites by conducting an NMR analysis. Table 1 tabulates the information obtained from the NMR results. The incorporation of 0.01 wt% and 0.05 wt% GO enhanced the hydration process of C_3_S by 68.2% and 72.4%, respectively, demonstrating GO’s beneficial effect on the hydration process.

#### 2.4.7. Mercury Intrusion Porosimetry (MIP)

MIP is a destructive method adopted to obtain the volume of pores, surface area of a material, total connected porosity, and pore size distribution. It has been commonly employed to study the microstructure of GRCCMs. The electrical and dielectric properties of GRCCMs are closely related to the size, distribution, and connectivity of pores in the composites. Because the pore structure determines the charge transport properties of GRCCMs, the testing and characterization of the pore structure are essential to provide information to improve the material properties of multifunctional GRCCMs.

The effect of the incorporation of rGO on the pore refinement of Portland cement was studied by Murugan et al. [102] via MIP. Table 2 tabulates the information obtained from the MIP results. The OPC paste with well-dispersed rGO was found to reduce the capillary pores by 32.1%. However, the rGO addition increased the gel pores by up to 36.5%.

**Figure 18:**
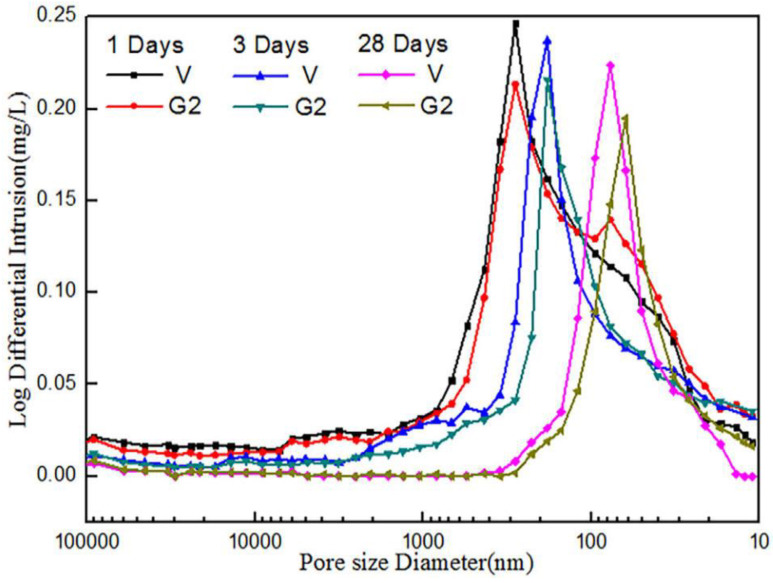
Pore size distribution of GNP/cement composites. Reprinted with permission from Ref. [55]. Copyright 2019 Elsevier.

Wang et al. [26,55,56,84] investigated the porosity and microstructure of GNP/cement composites by the MIP technique. Figure 18 shows the pore size distribution for different cement composite samples, in which V and G2 denoted pure cement samples and the sample with GNP reinforcement. The addition of GNP remarkably improved the pore structure of the CCMs. The average diameter and porosity of the reinforced CCMs decreased by up to 10% and 40%, respectively, after 28 days of curing when compared to the plain cement sample.

The MIP results by Du et al. [103,104,105] reveal that the critical size in CCMs can be reduced by more than 30% due to the addition of 2.5 wt% GNP. The nanoparticle size of GNP reinforcement can facilitate the nucleation for cement hydration products. Tao et al.’s [106] MIP investigation found that due to micro filling and nucleation provided by the addition of GNP, the porosity of the reinforced CCMs decreased. However, when the GNP concentration further increased, the porosity was increased because of agglomerations. Liu et al.’s [44] MIP results revealed that the incorporation of graphene enhanced the compactness and refined the microstructure of the CCMs.

#### 2.4.8. Scanning Electron Microscopy (SEM)

The dispersion of graphene filler in CCMs plays a crucial role in their mechanical and physical conductivity. SEM is an electron microscope that uses a focused beam of electrons to scan the surface of a sample. The dispersion of graphene filler in CCMs can be studied by SEM, and the morphological characteristics, structure, and defects of graphene filler can be observed.

**Figure 19:**
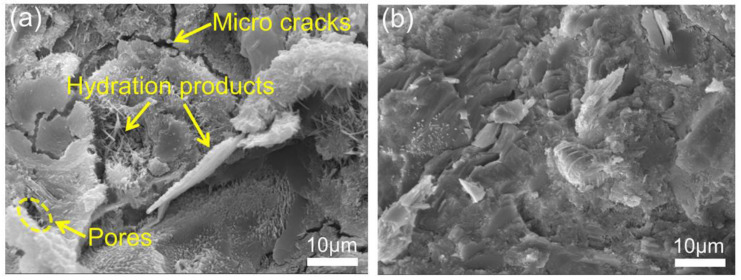
SEM images of cement paste after 28 days: (**a**) 0 wt% GO; (**b**) 0.6 wt% GO. Reprinted with permission from Ref. [107]. Copyright 2020 Elsevier.

Jing et al. [107] investigated the microstructures of plain cement and GO-reinforced cement samples by SEM (as shown in Figure 19). The image of the plain cement sample demonstrated that many micro-cracks and pores existed among the crystals. In contrast, with the addition of GO, there existed lots of calcium silicate hydrate gel and rod-shaped C_6_AS_3_H_32_ with no separated crystals being observed. The results showed that the samples with GO had a compact microstructure and fewer pores and cracks. Such a comparison of the SEM images suggested that the incorporation of the GO enhanced hydration and remarkably improved the microstructure of the cement sample. Alkhateb et al. [108] used SEM to have the surface topography and the composition of GRCCMs. Tong et al. [109] used GNP as fillers to reinforce CCMs. A nano-scale characterization, which was focused on the microstructure of the cement paste around the graphene reinforcements, was carried out to analyze the reinforcing mechanism. Pei et al. [110] modified CCMs by introducing high-quality graphene and PVA. By the analysis of the SEM images, it was found that graphene and cement were tightly bound in the composites.

#### 2.4.9. Friction Force Microscopy (FFM)

**Figure 20:**
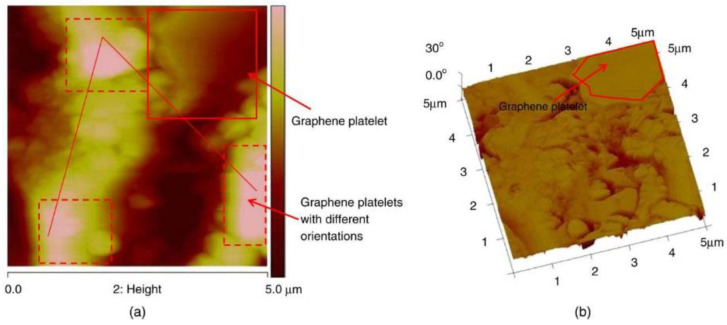
FFM images of GNP/cement composites: (**a**) Height image; (**b**) Phase image. Reprinted with permission from Ref. [108]. Copyright 2013 American Society of Civil Engineers.

Apart from SEM, FFM is another powerful tool for the investigation of the topology and morphology of GRCCMs. FFM is a type of scanning probe microscopy with a very high-resolution. It has been adopted as a technique to study the physical and chemical properties of GRCCMs. Horszczaruk et al. [22] used FFM to examine the early age mechanical response of the cement mortar modified with GO. From their topology images, the distributions of Young’s modulus for the two composite samples were estimated. Figure 20 shows the FFM image of GNP reinforced CCMs by Alkhateb et al. [108] The FFM images disclosed the C-S-H structures with both high-density and low-density. The phase image provided information on the stiffness variation of the specimen. The dark brown colour reflected the deep topography, while the bright pink colour reflected the high topography. Particularly, the image identified the GNP filler at the top-right corner. It was confirmed that the GNP correlated with the high-stiffness phase topology.

## 3. Electrical Property

It is evidenced that the addition of graphene fillers into the cement matrix significantly increases/decreases the electrical conductivity/resistivity of the reinforced composites. Such an increase in the electrical conductivity can significantly promote the application and sensitivity of such CCMs in detection of water/chloride ion penetration and corrosion occurred in the concrete structures. Table 3 summarizes the work on investigating the electrical properties of GRCCMs.

**Figure 21:**
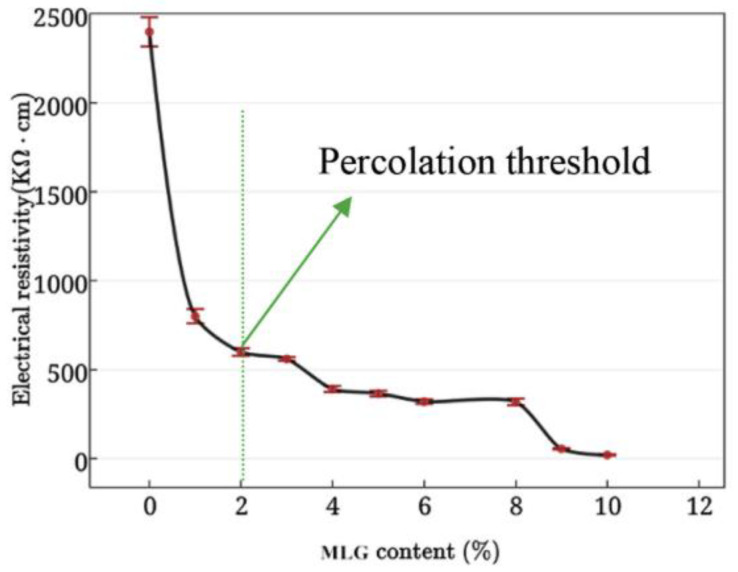
Electrical resistivity of graphene/cement composites. Reprinted with permission from Ref. [46]. Copyright 2017 Elsevier.

Bai et al. [43] measured the electrical conductivity of GRCCMs by the four-probe method. The experiments showed that the electrical conductivity was related to the distribution and concentration of the graphene fillers. As the graphene concentration exceeded the percolation threshold, the water content and curing age had limited effects on the electrical conductivity. Figure 21 shows the relationship between the electrical resistivity of the GRCCMs and the volume fraction of graphene measured by using DC (direct current) and AC (alternating current) methods [46]. When the graphene content was smaller than the percolation threshold, i.e., 2 vol%, the separation between the neighboring fillers was too far away and the graphene in the composites could not form a conductive network. Therefore, the electrical resistivity was still relatively high. As the graphene concentration further increased, the resistivity decreased.

**Figure 22:**
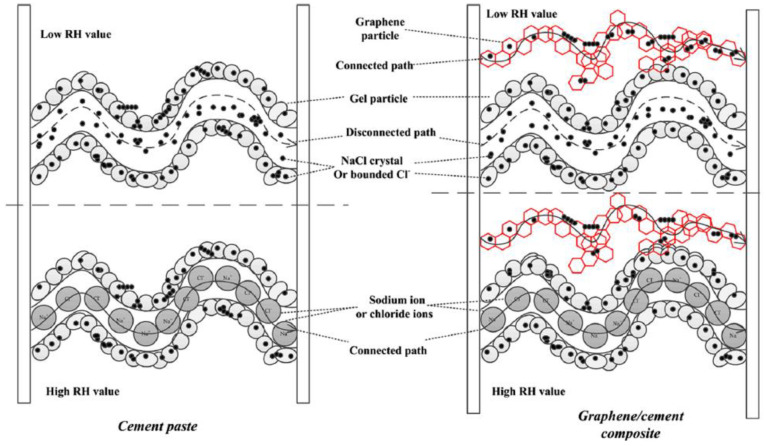
Formation of conductive paths in cement paste and GRCCMs. Reprinted with permission from Ref. [47]. Copyright 2017 Elsevier.

Jin et al. [47] fabricated a graphene-modified cement composite and utilized the electrical conductivity of composites to develop a non-destructive method to monitor the penetration of chloride ions in the concrete structure. Figure 22 shows the conductive paths formed between graphene fillers in the composites. Their results demonstrated that the electrical conductivity of the GRCCMs increased as the chloride concentration increased.

Zhang et al. [53] developed a novel self-sensing cement composite by adding rGO. The experiments indicated that the dispersion of the rGO increased the electrical conductivity by 23%. Goracci et al. [114] investigated the conduction mechanisms of GNP-reinforced CCMs. The reduction in electrical resistivity of the reinforced composites was attributed to the charge transport property and pore refinement by the addition of GNPs. Liu et al.’s [79] experiments showed that as the GNP concentration increased. The electrical resistance of the reinforcement CCMs had three zones, i.e., insulated, semi-conducted, and conducted zones. Sartipi et al. [74] studied the electrical resistivity of GO-reinforced CCMs. The bulk electrical conductivity of the sample was tested at 7, 14, and 28 days. It was evidenced that the addition of GO increased the electrical conductivity of the sample. Rehman et al. [113] found that with the incorporation of graphene, the electrical resistivity of CCMs was decreased by up to 67.8%.

**Figure 23:**
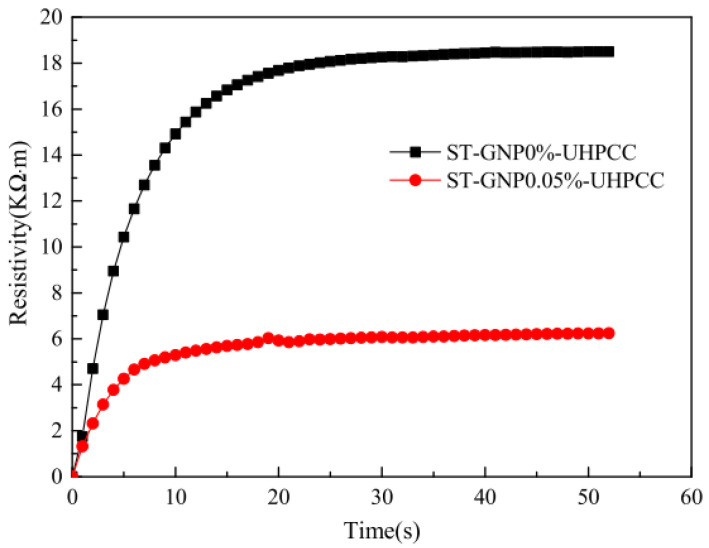
Variation of electrical resistivity with time. Reprinted with permission from Ref. [112]. Copyright 2019 Elsevier.

Sedaghat et al. [30] investigated the electrical conductivity of GRCCMs with different graphene concentrations, i.e., 0, 1 wt%, 5 wt%, and 10 wt%. They found a significant effect of the graphene fillers on increasing the electrical conductivity of the composites. Du et al. [115] reported that the change in electrical conductivity was greater than 1 order of magnitude with and addition of 15 wt% GNPs. Bai et al. [69] experimentally investigated the influences of silica fume concentration on electrical resistivity of the GRCCMs. Guo et al. [112] prepared high-performance CCMs by using GNP as reinforcing fillers. It was found that the introduction of GNPs into cement matrix reduced the resistivity of the CCMs from 18.85 kΩ·m to 6.26 kΩ·m (as shown in Figure 23). The increase in the resistivity of the GRCCMs can significantly improve the sensitivity of the CCMs when they are serving as sensors for structural health monitoring.

**Figure 24:**
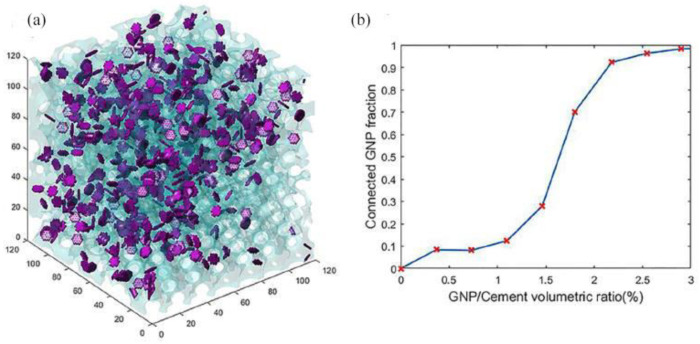
GNP/cement composites (**a**) Simulation model; (**b**) Simulation result. Reprinted from Ref. [72]. Copyright 2018 Elsevier.

Compared to experimental work, relatively fewer modeling studies have been found for the electrical conductivity of GRCCMs. Le et al. [111] added GNP into the cement matrix to prepare CCMs with electrical conductivity to quantify the material damage by measuring the change of electric potential. The authors used percolation theory to study the conductive behaviours of GRCCMs. It was found that the electrical conductivity of the composites suddenly increased remarkably when the graphene concentration reached the percolation threshold. Liu et al. [72] established a visual simulation model as shown in Figure 24 to analyze the percolation of GNP/cement composites. The percolation threshold was found to be 2.2 vol% in the simulation.

**Figure 25:**
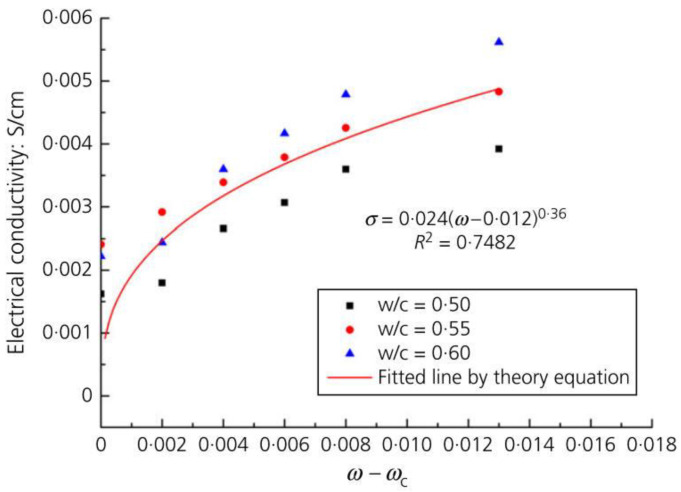
Experimental validation of percolation theory. Reprinted with permission from Ref. [43]. Copyright 2020 Thmas Telford Ltd.

Bai et al. [43] also employed percolation theory to study the conductivity of GRCCMs. The percolation theory was validated by experimental results (as shown in Figure 25). It was found that the percolation threshold did not depend on water/cement ratio. When the graphene concentration was smaller than the percolation threshold, the curing time had a considerable influence on the electrical conductivity of the composites. Once the conductive network was formed, the curing time had a limited effect on the electrical conductivity. As the graphene content increased, the influence of water on the conductivity of the composites increased. When the graphene concentration was smaller than the percolation threshold, the wet-state electrical conductivity was much higher than the dry-state conductivity. As the graphene concentration exceeded the percolation threshold, the conductivity of the dried composites was higher than that of the wet-state composites.

## 4. Piezoresistive Property

CCMs with piezoresistive properties have demonstrated great potential in developing smart civil engineering structures with the capability of self-sensing and structural health monitoring. For example, such CCMs with a piezoresistive property can be used in buildings and infrastructures to monitor the strain and crack in the structures [116,117]. Therefore, the improvement in the piezoresistive property of the GRCCMs can significantly enhance the sensitivity of such multifunctional composites as sensors.

It is evidenced that the electrical conductivity/resistivity possessed by GRCCMs can vary with the deformation of the materials and structures. Extensive studies have been done on the piezoresistive property of GRCCMs. Table 4 summarizes the work on investigating the piezoelectrical properties of GRCCMs.

**Figure 26:**
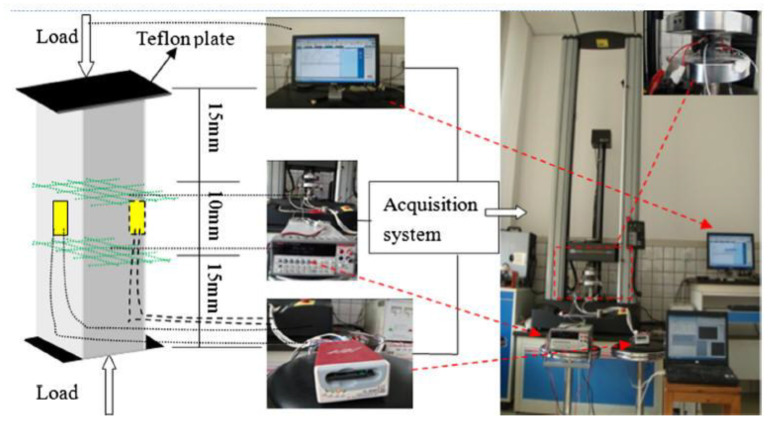
Sketch of measuring piezoresistivity of GRCCMs. Reprinted with permission from Ref. [121]. Copyright 2017 Elsevier.

Du et al. [115] studied the piezoresistivity of CCMs incorporated with GNPs by the four-probe testing method. A gauge factor in the order of magnitude of 10^2–^10^3^ was obtained. It was found that the gauge factor increased with the increase of GNP concentration at low strain. Such observation was attributed to the balance between the contribution of the piezoresistivity from the matrix and the interface between matrix and GNP fillers. Liu et al.’s [79] experiments investigated the piezoresistivity of GNP and graphene oxide nanoplatelet (GONP) reinforced CCMs. GNPs were found to be a better reinforcing filler than GONP and the cement mortar with a concentration of 6.4% GNP had better performance in the piezoresistive property. Rehman et al. [81] tested the piezoresistive properties of GNP/cement composites to obtain the self-sensing attribute. A drop of 42% in electrical resistivity of the composites was observed at the maximum compressive load. This variation of the electrical resistivity with compression was used for detecting damage and crack propagation in CCMs. Sun et al. [121] investigated the piezoresistive properties of CCMs filled with nano graphite platelets. The electrical response of the CCMs were subjected to cyclic compressive stress under different loading conditions. Figure 26 shows the set up for testing the piezoresistivity of the composites. It was found that the composite without or with a low concentration of the reinforcements had no or unobvious piezoresistive effects. The composites with a concentration of 5 vol% reinforcement had the most sensitive piezoresistivity.

**Figure 27:**
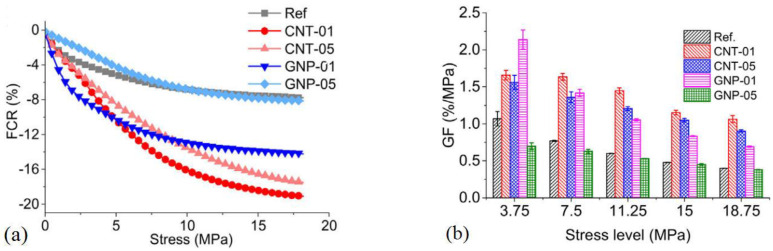
Piezoresistive results of cement composites: (**a**) Fractional change ratio of electrical resistivity of GNP and CNT reinforced samples; (**b**) Gauge factor of GNP and CNT reinforced samples. Reprinted with permission from Ref. [122]. Copyright 2020 Elsevier.

Tao et al. [122] compared the performances of GNP- and CNT-reinforced CCMs. Figure 27 shows the change ratio of electrical resistivity and gauge factor with stress. The cement composite sample containing 0.1% GNP (GNP-01) was found to have a more significant change ratio of electrical resistivity and a larger gauge factor compared to the other samples. This indicated that GNP-01 was more sensitive to stress and had a better piezoresistive performance.

**Figure 28:**
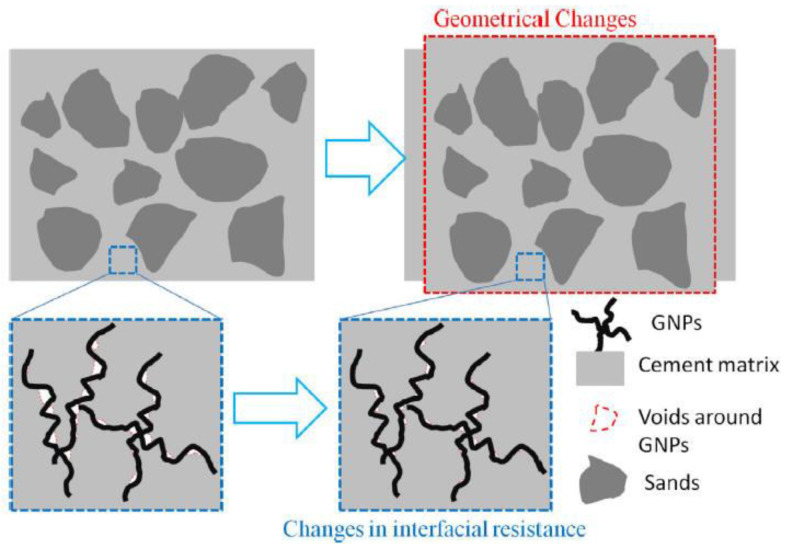
Schematic diagram of mechanisms for piezoresistive responses of GNP-modified cement composites. Reprinted with permission from Ref. [106]. Copyright 2019 Elsevier.

Based on the analogy between electric field and the electrostatic field under anti-plane shear loading, Pang et al. [118] confirmed the potential for piezoresistive strain sensing of CCMs reinforced by GNPs. Guo et al. [112] prepared high-performance CCMs by using GNP as reinforcing fillers. It was found with the introduction of GNPs into the cement matrix, the self-sensing performance of the composites with 0.05% GNPs was almost doubled compared to the composites without GNPs. Pei et al. [110] prepared graphene/PVA hybrid modified CCMs and their electrical and piezoelectric properties were significantly improved with a low concentration of graphene filler. Roopa et al. [123] developed smart cement composite sensors by using different kinds of conductive filler (CNTs, CF, and graphene). The electromechanical examination of the fabricated sensor incited good strain sensing with respect to the applied load. Tao et al. [106] investigated the GNP reinforced CCMs. As shown in Figure 28, the piezoresistivity of the composites under cyclic compression was quantitatively evaluated by the four-probe method. Results showed that the variation of the electric conductivity with GNP concentration showed a percolation behaviour. The piezoresistive attributes had very limited dependence on the loading levels and the GNP concentration. The mechanisms that underpin the piezoresistive reaction were attributed to the interfacial conductance between GNPs and cement matrix and elastic deformation.

**Figure 29:**
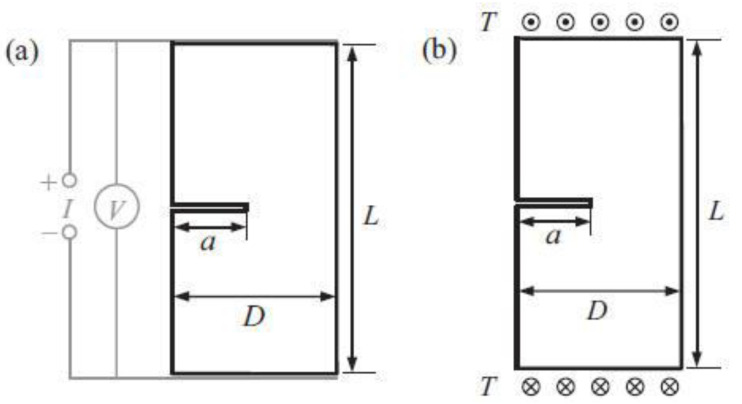
Sketch of damaged specimen with a notch. (**a**) Crack detection with conductive plates attached to the ends of the specimen; (**b**) Mathematical analogy of anti-plane shear applied to the ends of the specimen. Reprinted with permission from Ref. [111]. Copyright 2014 Elsevier.

Compared to the plain cement sample, Zhu et al. ’s [77] study showed that the dispersion of graphene remarkably decreased the resistivity with enhanced pressure sensitivity. The reinforced CCMs showed improved piezoresistivity when the graphene concentration was 0.05 compared to the case with 0.5 wt% graphene. Madbouly et al. [124] experimentally explored the piezoresistivity of the GO reinforced CCMs. Based on the obtained results, the composites could be efficiently adopted as piezoresistive sensors. Apart from experiments, some theoretical work has been done on the piezoresistive property of GRCCMs. Under anti-plane shear loading using the similarities between the 2D electrostatic and electrostatic fields (as shown in Figure 29), Le et al. [111] developed a mathematical model to correlate the damage extent to the change of electrical resistance. The model was validated by their experimental results.

## 5. Electromagnetic Property

With the high demand for protecting the sensitive environment from radiation hazards and wireless communications, the electromagnetic interference (EMI) properties of the cement structure is getting more attention. Extensive results have evidenced that the addition of graphene fillers into cement materials is a promising method to develop multifunctional cement composite materials and structures with EMI attributes. Table 5 summarizes the work on investigating the electromagnetic properties of GRCCMs.

**Figure 30:**
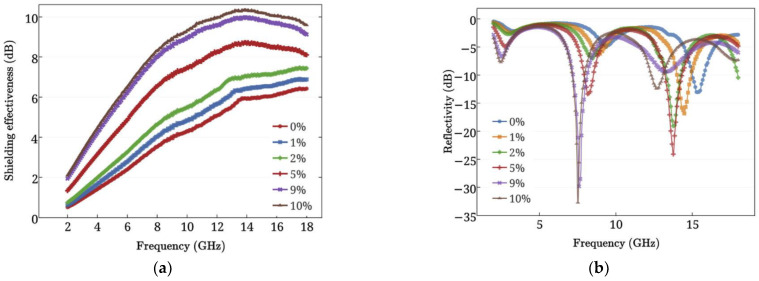
(**a**) Shielding effectiveness and (**b**) Reflectivity of GRCCMs. Reprinted with permission from Ref. [46]. Copyright 2017 Elsevier.

Figure 30 shows the variation of the shielding effectiveness and reflectivity with the frequency of GRCCMs by Sun et al. [46] It was found that as the graphene filler content increased, the shielding effectiveness of the modified CCMs increased while the reflectivity decreased. Compared to plain cement, the shielding effectiveness and the reflectivity was increased by up to 1.6 and 7 times, respectively.

**Figure 31:**
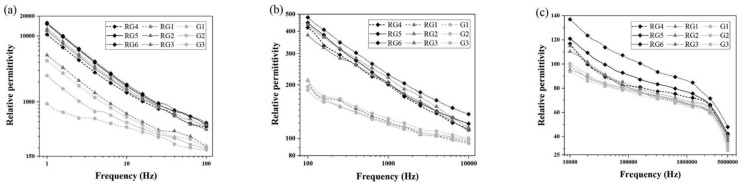
Relative permittivity of cement composites with different GO concentrations within different frequency ranges (**a**) 1–10^2^ Hz; (**b**) 10^2^–10^4^ Hz; (**c**) 10^4^–5×10^6^ Hz. Reprinted with permission from Ref. [127]. Copyright 2019 Elsevier.

Goracci et al. [114] investigated the electromagnetic properties of GNP-reinforced CCMs through dielectric spectroscopy within the frequency range 10^−2^ Hz–10^6^ Hz. After the addition of GNP, the charge transport properties were enhanced, and the electrical capacitance had higher values over all the frequency ranges involved. Sun et al. [129] added rGO, nano ferroferric oxide, and nano nickel particles into the cement. With 0.05% rGO added, the minimum reflectivity of the composite reached −14.7 dB at 2.15 GHz. When the reflectivity is smaller than −5 dB, an effective bandwidth of 14.4 GHz was achieved. Chen et al. [126] dispersed GO and CF into cement and found that this combination was more effective in improving EMI shielding than CF only. With 0.4 wt% GO-CF, a shielding effectiveness of 34 dB was achieved within the frequency range 8.2–12.4 GHz, which was 31% higher compared to the value with CF only. Singh et al. [125] prepared GO-ferrofluid-cement composites to test the EMI effectiveness within the frequency range 8.2–12.4 GHz. It is shown that the addition of 30 wt% GO and ferrofluid in the cement matrix resulted in a shielding effectiveness of 46 dB. Cui et al.’s [132] experiments demonstrated that by adding 5% graphite platelets, the electromagnetic wave reflectivity of the composites was decreased by 38% compared to pure CCMs. Long et al. [127] studied the combined effect of waste cathode-ray tube (CRT) and GO on mitigating electromagnetic interference for CCMs (as shown in Figure 31). The combined effect between waste CRT glass and GO significantly improved the permittivity.

**Figure 32:**
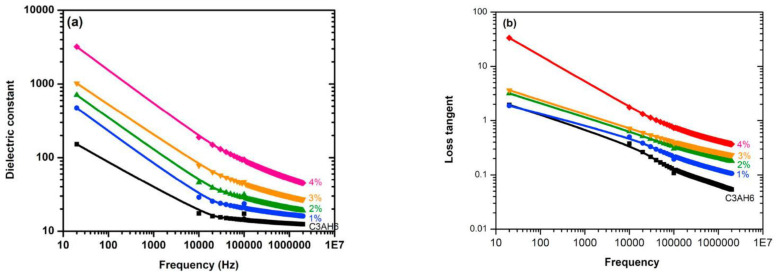
(**a**) Dielectric constant and (**b**) dielectric loss of CCMs with different concentrations of rGO. Reprinted with permission from Ref. [31]. Copyright 2019 Elsevier.

Mazzoli et al. [130] dispersed GO and metallic fibers into a cement matrix and investigated the EMI shielding properties. It was found that the use of GO significantly improved the EMI shielding effectiveness. Lv et al. [128] prepared CCMs reinforced with GNP and hollow glass microspheres and examined their electromagnetic wave absorbing properties. The reinforced CCMs had significantly improved absorbing properties. Within the range of 2–18 GHz, the average reflectivity loss was –8.2 dB and the bandwidth was 4.4 GHz below –5 dB. Zhao et al. [131] experimentally investigated the EMI properties of the prepared GO reinforced CCMs at 8.2–12.4 GHz. It was found that GO was essential in improving EMI effectiveness due to the absorption of electromagnetic radiation. Khushnood et al.’s [133] experimental results also demonstrated the effectiveness of enhancing the EMI properties through dispersing graphene into a cement matrix. Phrompet et al. [31] investigated the dielectric constant of rGO-reinforced CCMs (as shown in Figure 32). These results confirmed that the reinforced CCMs were effective in enhancing dielectric properties, demonstrating great potential as electromagnetic shielding material candidates.

## 6. Conclusions

In this review, the graphene fillers and the cement matrix used to develop high-performance and multifunctional CCMs are firstly summarized. The advantages and disadvantages of different graphene fillers and cement matrices are discussed. It was found that compared to graphene, GO and rGO, GNP demonstrated great potential for practical engineering application due to their comparative material properties with moderate cost and availability for mass production. Methods/techniques, including TG/DTG/DSC, FTIR, XPS, XRD, Raman spectroscopy, NMR, SEM, TEM, and FFM for characterizing GRCCMs are introduced and discussed. Then the present study introduces experimental and theoretical studies on three material properties of GRCCMs, i.e., electrical conductivity/resistivity, piezoresistivity, and electromagnetic interference. From the above review, it is demonstrated that the dispersion of graphene fillers can increase the hydration process of the composites and significantly improve the three material properties as involved in this paper, which enable such modified CCM promises in developing self-sensing and smart civil engineering materials and structures. However, there are still challenges in developing GRCCMs. For example, more methods and work need to be explored for the good dispersion of graphene fillers into a cement matrix for large scale civil engineering structures. Moreover, although extensive work has been done on graphene/cement composites, the majority of the work is focused on experiments and very limited theoretical studies can be found. Therefore, in the future, more theoretical work may need to be conducted for a more comprehensive and deeper understanding of the mechanisms that underpin the influences of graphene fillers on the material properties of the CCMs involved.

## Figures and Tables

**Table 1:** Results obtained from ^29^Si MAS NMR spectroscopy. Reprinted with permission from Ref. [101]. Copyright 2019 Elsevier.

GO Content (wt%)	Q^0^ (%)	Q^1^ (%)	Q^2^ (%)	Q^2^/Q^1^	MCL	α (%)
0.00	45.20	27.02	27.78	1.03	4.056	54.8
0.01	32.12	36.46	32.42	0.89	3.778	68.2
0.05	27.65	39.98	32.37	0.81	3.619	72.4

**Table 2:** Pore structure feature of rGO-reinforced cement paste. Reprinted with permission from Ref. [102]. Copyright 2016 Elsevier.

Paste	Gel Pores <10 nm in mm^3^/g	Capillary Pores (10 nm–10 μm) in mm^3^/g	Threshold Diameter in nm	Most Likely Diameter in nm
Control	10.69	48.58	26.8	14.7
rGO	14.60	33.01	20.7	12.9
n-Al_2_O_3_	26.97	60.58	39.1	27.1
n-SiO_2_	23.42	57.76	28.6	20.4

**Table 3:** Summary of studies on electrical properties of GRCCMs.

Filler Type	Matrix	Preparation	Electrical Properties	Ref.
Graphene	Paste	Dry-mix	The addition of 1 vol% of graphene enhanced the electrical conductivity by 3 orders. With 10 vol% graphene, the conductivity was 10^−2^ S/m.	[30]
Graphene	Paste	Wet-mix	The electrical conductivity of the composites demonstrated S-shaped curves.	[43]
Graphene	Paste	Wet-mix	The percolation threshold for electrical resistivity of the composites was close to 2 vol%.	[46]
rGO	Paste/Mortar	Wet-mix	The electrical conductivity of rGO/cement paste was increased by 23%.	[53]
GO	Paste	Wet-mix	The cement paste with 0.08 wt% GO demonstrated much lower electrical resistivity.	[58]
GNPs	Mortar	Dry-mix	The electrical resistivity of GNP/cement composites with 2.0 vol% GNP was lowered to 100.8 Ω·cm.	[72]
rGO	Mortar	Wet-mix	When the rGO content was 2.00 wt%, the electrical resistivity of the sample dropped by 40%.	[88]
GO/rGO	Paste	Wet-mix	The electrical resistivity had highest value at 0.02 wt% GO and rGO composites then it reduced as the concentration further increased.	[93]
GNPs	Mortar	Wet-mix	When 1 wt% GNP is dispersed, the electrical resistance was reduced from 300 kΩ to 19 kΩ.	[106]
GNP	Mortar	Wet-mix	The electrical resistivity showed a decrease of more than 1 order of magnitude when 2.4 vol% GNP was added.	[111]
GNP	Paste	Wet-mix	The introduction GNPs was confirmed to decrease the electrical resistivity of cement paste from 18.85 kΩ·m to 6.26 kΩ·m.	[112]
Graphene	Paste	Wet-mix	The electrical resistivity of the cement composite sample was decreased by 67.8% by adding graphene.	[113]

**Table 4:** Summary of studies on piezoelectrical properties of GRCCMs.

Filler Type	Matrix	Preparation	Piezoelectrical Properties	Ref.
rGO	Paste/Mortar	Wet-mix	For paste composites, the pressure sensitivity and strain sensitivity were 2.52%/MPa and 363.10, respectively when the rGO concentration was 1.0 wt%. For mortar composites, the pressure sensitivity and strain sensitivity were 1.28%/MPa and 147.80, respectively, when the rGO concentration was 2.00 wt%.	[53]
GNPs	Mortar	Wet-mix	The mortar with 6.4 wt% GNPs had the best piezoresistive performance.	[79]
GNPs	Concrete	Wet-mix	Applying compressive loading, the resistivity value was reduced by 42%.	[81]
rGO	Mortar	Wet-mix	The largest strain sensitivity coefficient and stress reached up to 1.28%/MPa and 147.80, respectively, with 2.00 wt% rGO.	[88]
GNPs	Paste	Wet-mix	The resistance of the GNP/cement composite monotonously increased and decreased under cyclic compressive stress. The resistance could go back to the initial state when the pressure was zero.	[112]
GNP	Mortar	Wet-mix	When the compressive strain was larger than 400 microstrain, the gauge factor was 100 after percolation.	[118]
GNP	Mortar	Wet-mix	Subjected to vertical compression, the electrical resistances in vertical, horizontal, and diagonal directions dropped by 5.5%, 1.8%, and 6.7%, respectively.	[119]
GNP	Mortar	Wet-mix/Dry-mix	The increase of GNP concentration from 7.5 wt% to 10 wt% deteriorated the gauge factor. There existed an optimum GNP concentration providing the best self-sensing properties.	[71]
Graphene	Mortar	Wet-mix	Under maximum loading, the electrical resistance variation ratio was 2% and 25% for mesh and wire probes, respectively.	[120]

**Table 5:** Summary of studies on electromagnetic properties of GRCCMs.

Filler Type	Matrix	Preparation	Electromagnetic Properties	Ref.
Graphene	Paste	Wet-mix	Shielding effectiveness and wave absorption were increased by 1.6 and 7 times, respectively.	[46]
rGO	Mortar	Wet-mix	Shielding effectiveness was improved by 45%.	[53]
rGO	Mortar	Wet-mix	Shielding effectiveness was increased by 30%~45%.	[88]
GO	Paste	Dry-mix	Shielding effectiveness could reach up to 46 dB.	[125]
GO	Mortar	Wet-mix	Shielding effectiveness was increased by 31%.	[126]
GO	Mortar	Wet-mix	Relative permittivity was increased by about 50% and 200% when the frequency is in the ranges of 10^4^–5 × 10^6^ Hz and 10^1^–10^3^ Hz, respectively.	[127]
GNP	Paste	Wet-mix	Within the range of 2–18 GHz, the average reflectivity loss was –8.2 dB and the effective absorption bandwidth was as high as 4.4 GHz.	[128]
rGO	Paste	Wet-mix	Within the range of 1–18 GHz, a minimum reflectivity of –14.7 dB was achieved. An effective bandwidth of 14.4 GHz was achieved when the reflectivity was smaller than –5 dB.	[129]
GO	Mortar	Wet-mix	Shielding effectiveness reached up to 40–50 dB.	[130]
GO	Paste	Wet-mix	It was found that the contribution to the increase of shielding effectiveness came from the absorption growth rather than reflection.	[131]

## Data Availability

Not applicable.
